# The mPower (Mother’s Power) Initiative: Improving Health Behavior Through Peer Support and Health Literacy for Mothers of Children with Cerebral Palsy in Rural Bangladesh

**DOI:** 10.3390/children11121438

**Published:** 2024-11-26

**Authors:** Genevieve Perrins, Israt Jahan, Md. Nuruzzaman Khan, Mahmudul Hassan Al Imam, Rosalie Power, Catherine King, Mohammad Muhit, Nadia Badawi, Gulam Khandaker

**Affiliations:** 1 Central Queensland Public Health Unit, Rockhampton, QLD 4700, Australia; genevieve.perrins@health.qld.gov.au (G.P.);; 2 Faculty of Medicine and Health, University of Sydney, Camperdown, Sydney, NSW 2050, Australia; 3 CSF Global, Dhaka 1212, Bangladesh; 4 School of Health, Medical and Applied Sciences, CQ University, Rockhampton, QLD 4701, Australia; 5 Department of Population Science, Jatiya Kabi Kazi Nazrul Islam University, Trishal, Mymensingh 2220, Bangladesh; mdnuruzzaman.khan@uon.edu.au; 6 Translational Health Research Institute, University of Western Sydney, Campbelltown, NSW 2751, Australia; 7 Sydney School of Public Health, University of Sydney, Camperdown, Sydney, NSW 2050, Australia; 8 Grace Centre for Newborn Intensive Care, The Sydney Children’s Hospitals Network, Westmead, NSW 2145, Australia; 9 Cerebral Palsy Alliance Research Institute, Sydney Medical School, The University of Sydney, Camperdown, NSW 2050, Australia

**Keywords:** cerebral palsy, children with disability, mother, caregiver, empowerment, health knowledge, support group, low- and middle-income country, Bangladesh

## Abstract

Background/Objectives: Cerebral palsy (CP) affects a substantial number of children, particularly in low- and middle-income countries such as Bangladesh. Maternal health literacy is critical to the health and well-being of children with CP, particularly in low-resource settings. In this study, we sought to assess how the mPower (mother’s power) community-based intervention impacted mothers’ CP-specific knowledge, as well as their utilization of rehabilitation services in rural Bangladesh. Methods: This quasi-experimental study was conducted with a group of mothers of children with CP, formed through the ongoing initiatives of the Bangladesh CP Register in rural Bangladesh. A pre-post-intervention comparison method was used to assess the outcomes of the intervention. Results: Mothers who participated in over two-thirds of the mPower sessions demonstrated a significant increase in CP-related knowledge (75.5% vs. 63.6%, *p* = 0.04). Additionally, mothers who attended two-thirds of the mPower sessions utilized rehabilitation services more often compared to those who attended fewer sessions (55.3% vs. 22.6%, *p* < 0.001). Conclusions: The mPower intervention successfully improved health literacy and likely increased rehabilitation service utilization among mothers of children with CP in rural Bangladesh.

## 1. Introduction

Caused by abnormal development or injury to the developing brain, cerebral palsy (CP) is a condition that can lead to a range of motor impairments including difficulty with movement, posture, and coordination. CP also has a number of comorbidities, including but not limited to epilepsy, intellectual disability, and vision, hearing, and speech problems [1,2]. It is a lifelong condition, often requiring dedicated support. Globally, CP is the most common form of childhood physical disability; however, a significant portion of this burden occurs in low- and middle-income countries such as Bangladesh, where the estimated prevalence is as high as 3.4 per 1000 live births [3]. By this account, there are approximately half a million parents and caregivers of children with CP in Bangladesh.

Health literacy is defined by the World Health Organization as the “cognitive and social skills which determine the motivation and ability of individuals to gain access to, understand and use information in ways which promote and maintain good health” [4]. When an individual is unable to make their own health decisions due to social pressures, reliance on healthcare professionals, age, or cognitive limitations, the health literacy of a primary caregiver becomes essential for ensuring overall health and well-being [5].

Globally, there is a dearth of literature relating to the health literacy of primary caregivers of children with CP in LMICs [6]. However, low maternal health literacy can significantly impact health outcomes for children with CP by hindering effective management and care. Particularly in low-resource settings like Bangladesh, where exposure to health practitioners is sparse, children rely on their mothers to fill the void in the health system—making her embody the mother, doctor, coordinator, therapist, and nutritionist [7]. However, when mothers have limited understanding of medical information, treatment options, and care strategies, they may struggle to make informed decisions regarding their child’s health and well-being. This lack of comprehension can result in delays in seeking appropriate medical care, difficulties in adhering to treatment regimens, and challenges in implementing recommended therapies and interventions [7]. Consequently, children with CP may experience suboptimal health outcomes, including worsened physical and developmental progress, increased complications, and reduced overall quality of life. Enhancing maternal health literacy through education and support can therefore play a crucial role in improving health outcomes for these children.

In Bangladesh, rehabilitation service uptake is very low; according to [8], only 50.2% of children with CP in rural Bangladesh had ever received rehabilitation therapy at the time of writing. A lack of awareness regarding availability and value of rehabilitation services was reported by 84.2% of parents whose children had never accessed services in rural Bangladesh [8]. While the reasons for low uptake are multi-factorial, there are significant associations with maternal and paternal educational status, monthly household income, and disease severity [8]. Therefore, there is an urgent need to address parental gaps in knowledge regarding the availability of services, as well as an opportunity to impart on parents the importance of timely and consistent rehabilitation in managing CP.

Peer-to-peer support groups are increasingly being recognized as a viable solution to addressing informational gaps in caregivers which impact health outcomes in children [9,10,11,12]. Peer-to-peer support has been shown to be effective in reducing stigma and isolation, improving mental health, increasing access to and utilization of health services, enhancing engagement and motivation, and leading to greater empowerment [13]. These benefits underscore the importance of community-driven support mechanisms in mediating the impact of disjointed health services.

Established in 2015, the Bangladesh CP Register (BCPR) is a comprehensive surveillance program designed to collect and manage information on children diagnosed with CP in the rural region of Sirajganj, Bangladesh. It aims to provide a detailed and systematic record of CP cases across the region, facilitating better understanding, research, and management of the condition. As part of this operation, mothers of children with CP began to organically form groups to connect over their shared experiences. The study team saw this as an opportunity to formalize these groupings, establishing an innovative and replicable model of service which could be observed. We used content from the expertly developed and fit-for-purpose Getting to Know CP manual to systematically impart knowledge on mothers in areas such as positioning, epilepsy, and using assistive devices [14]. The mPower (mothers’ power) peer-to-peer support group was implemented as part of a post-intervention comparison study.

In this study, we aimed to evaluate the impact of mPower intervention on mothers’ empowerment by enhancing their knowledge about CP and utilizing rehabilitation services for their children in rural Bangladesh.

## 2. Materials and Methods

This was a quasi-experimental study conducted among a cohort of mothers of children with CP established as part of the ongoing activities of the Bangladesh CP Register (BCPR) in rural areas of Bangladesh. A pre-post-intervention comparison approach was adopted to measure intervention outcomes.

### 2.1. Ethical Consideration

Ethics approval was obtained from the Human Research Ethics Committee of the Bangladesh Medical Research Council (BMRC; approval number: BMRC/NREC/2019-2022/36).

### 2.2. Study Setting and Participants

The study was conducted in four sub-districts (i.e., Shahjadpur, Belkuchi, Ullahpara, and Sirajganj Sadar) of Sirajganj, Bangladesh. Mothers of children with CP registered in the BCPR in the study area were eligible to participate. Participation was voluntary. All eligible mothers were invited to participate in the study and those who provided consent were included in the study (i.e., study cohort). Participants were advised in detail about the process of participation, specifically that they would be allocated to groups according to geography. Information collected about mothers and their child by BCPR (including sensitive medical information) was not distributed to group leaders; instead, if mothers were comfortable, they were able to share this information directly.

### 2.3. Intervention Details (i.e., mPower Group)

As part of the study, we formed peer-to-peer support groups—called mPower—with an aim to empower mothers with the knowledge and skills to provide the best care possible for their children. Moreover, the mPower group emerged as a carer-led activity, in which mothers of BCPR sought out social connections.

We formalized a model around this initiative, setting down mPower as a potentially innovative model of care for children with CP. The aims of the intervention were to (i) increase mothers’ health literacy and self-efficacy by providing education on managing their child’s condition and (ii) provide a mechanism for social support for mothers of children with CP. Details of the mPower intervention have been published in one of our earlier publications [15].

### 2.4. Group Structure

Each mPower group comprised 8–10 mothers of children with CP living in the same locality. Participants were assigned to groups according to location, using demographic data collected as part of the BCPR. Each group is led by a group leader, who assisted the community mobilizers (CMs—paid project staff, BCPR) in identifying convenient meeting locations (often the yard of a member’s household) for group members. CMs, with support from group leaders, also coordinated activities and addressed barriers to running the sessions.

### 2.5. Session Structure and Contents

The group members met face-to-face, twice a month over 12 months of the intervention period, with each session lasting an average of 60 min. These sessions were organized to discuss different aspects of supporting children with CP in their daily activities. The group discussions were led by group leaders, with active participation of the peers/group members and supervision from CMs. BCPR used two CMs to manage and coordinate the program and attend sessions sporadically to support where required. Group leaders received intensive group training over 2 days on the Bangla-translated version of the “Getting to Know Cerebral Palsy” manual, which was then used to facilitate the discussion and as session resource material [14]. The manual was developed in collaboration with a Bangladeshi not-for-profit (CSF Global), making it specific to the cultural context. The manual consists of ten modules covering introduction to cerebral palsy, evaluating development and epilepsy, positioning, and carrying techniques, as well as addressing communication challenges, daily activities, feeding support, the importance of play, understanding disability in the community, organizing parent support groups, and using assistive devices to improve daily life. Sessions were delivered chronologically according to the 10 modules within the manual. The group meetings also provided a safe space for members to share their concerns and personal challenges and discuss their need for support in protecting both themselves and their children from any potential harm. Further details about session structure are available in our previous publications [15].

### 2.6. Follow-Up Period and Data Collection

Quantitative data were collected at baseline (i.e., pre-intervention/joining mPower group) and endline (post-intervention/12 months following joining the mPower group). The following data were collected and used in the study:

1. Socio-demographic characteristics: age of the participating mother/caregiver, educational level of the mother/caregiver and their partner, occupation of the mother/caregiver and their partner, age and sex of their child with CP, type of accommodation, household size, and monthly family income. These were used as baseline data to describe the cohort profile for this study.
2. Group meeting details (e.g., date, location, and attendance) and number of sessions attended by each participant.
3. Knowledge about CP: The participants (i.e., mPower group members) were interviewed pre- and post-intervention. A Bangla-translated and validated Knowledge of Cerebral Palsy Questionnaire (KCPQ) developed by Dambi et al. was utilized [16]. The questionnaire had 20 questions targeting knowledge assessment about etiology, stigma around causes of CP, clinical and motor features, and management.
4. Frequency and type of rehabilitation service utilized for their children with CP at the local facilities throughout the intervention period.

### 2.7. Data Analysis

Descriptive statistics (mean with standard deviation (SD), median with interquartile range (IQR), frequency with percentage) were used to describe the participant characteristics. The knowledge score was calculated using the method described by Dambi et al. [16], where each correct answer earned 1 point, and each incorrect answer earned 0 points. With 20 questions in total, the cumulative score ranged from 0 to 20. Bivariate analysis (cross tabulation with chi-squared test and logistic regression) was used to identify factors influencing mPower group attendance, improvement in CP-related knowledge and health/rehabilitation service utilization of their children. Odds ratio (OR) and adjusted OR (aOR) with 95% confidence intervals (CI) were reported. A *p* value <0.05 was considered statistically significant. All analyses were performed using Stata version 15.1.

## 3. Results

Among eligible mothers, *n* = 313 participated in the baseline knowledge assessment survey. Of those *n* = 289 completed the endline survey. The subsequent results section report findings from those *n* = 289 mothers/caregiver of children with CP in study area.

### 3.1. Socio-Demographic Characteristics

Overall, 65.7% of the participants and 68.9% of their partners had primary level education or lower, respectively. Most participants (95.5%) were housewives, and 40.2% of their partners were daily wage earners. For 61.0% of participants, their monthly income was less than $84 USD per month. The majority had a “kutcha” house (i.e., mud house or temporary house). Almost half (41.1%) had children with CP aged 0–5 years. Further demographic details are outlined in Table 1.

### 3.2. Participants Attendance to mPower Sessions and Improvement of Knowledge About CP

A total of 25 mPower sessions were conducted during the intervention period, and the average (SD) number of sessions attended by each participant was 9.5 (6.4), and 32.5% had attended more than two-third of the mPower sessions. 67.5% showed improvement in CP-related knowledge post-intervention (i.e., at end-line).

### 3.3. Factors Related to Improvement in CP-Related Knowledge of the Participating Mothers

Mothers who attended more than two-thirds of the mPower sessions showed a significantly greater improvement in CP-related knowledge than those who attended less (63.6% vs. 75.5% for mothers who had attended two-third vs. more than two-third mPower sessions, *p* = 0.04). A positive relation was also found between the improvement in mothers’ knowledge and both the household size and family’s monthly income (*p* = 0.03 for both). However, mothers’ education was not statistically significant in improving CP knowledge (*p* = 0.11) (Table 2).

### 3.4. Factors Related to Rehabilitation Service Utilization for Children with CP

Mothers who attended more than two-thirds of the mPower sessions used rehabilitation services more frequently than those who attended fewer sessions (55.3% vs. 22.6, *p* < 0.001). In addition to that, mothers who had completed secondary education or higher had a significantly higher rate of rehabilitation service utilization for their children with CP than those who completed primary education or lower (32.9% and 65.4% vs. 28.9%, *p* = 0.001). A similar pattern was observed for their partners education level (*p* = 0.003). Although not statistically significant (*p* = 0.39), an improvement in CP-related knowledge among mothers was positively related to increased rehabilitation service use for their children. (Table 3).

### 3.5. Predictors of Mothers’ Attendance at mPower Sessions, Improvement in CP Related Knowledge and Rehabilitation Service Utilization

Table 4 presents the results of the adjusted analysis, highlighting the predictors of attendance at mPower sessions, improvements in CP-related knowledge, and utilization of rehabilitation services. Attendance at mPower sessions was significantly influenced by the educational level of both mothers and their partners. Mothers with primary education or lower were 3.4 times more likely to attend than those with higher education (95% CI: 1.0, 11.3), while higher partner education increased the likelihood of attendance (aOR 3.8, 95% CI: 1.3, 11.7). Improved CP-related knowledge was linked to low maternal education, larger household size, and higher session attendance, with odds of improvement being 4.5 times (95% CI: 1.3, 15.5), 1.7 times (95% CI: 1.0, 3.0), and 1.9 times (95% CI: 1.0, 3.4) higher, respectively. Rehabilitation service utilization was 4.7 times higher among frequent session attendees (95% CI: 2.6, 8.5), but no significant relationship was found between improved knowledge and healthcare service use. Mothers with lower education levels had significantly lower odds of using rehabilitation services (aOR 0.2, 95% CI: 0.0, 0.6).

### 3.6. Factors Influencing Attendance of the Mothers at mPower Sessions

A higher rate of participation in mPower sessions was observed among mothers whose partners had completed secondary or higher-level education compared those whose partners had completed primary or lower education (42.4% and 45.6% vs. 27.1% respectively, *p* = 0.014). The contrary was observed for participants’ (i.e., mothers) education level; however, this was not statistically significant (*p* = 0.69). See Supplementary Table S1.

Overall, 67.5% of attendees showed improvement in disease-specific knowledge at endline and this was substantially higher for mothers who attended more than two-thirds of the mPower sessions (75.5%). Attendance at mPower was significantly influenced by educational attainment of both mother and father. In particular, mothers with lower levels of educational attainment were more likely to attend, compared with fathers where higher educational attainment had positive influence on maternal attendance. Mothers who frequented mPower sessions were 4.7 times more likely (95% CI: 2.6, 8.5) to access rehabilitation services; however, this was not significantly linked with knowledge gains.

## 4. Discussion

The aim of this study was to assess the impact of the mPower intervention on empowering mothers by increasing their knowledge about CP and improving their utilization of rehabilitation services for their children in rural Bangladesh. The findings of our study demonstrate the positive impact of peer-support interventions, such as mPower, on improving health literacy and rehabilitation service use among mothers of children with CP in rural Bangladesh. Mothers who attended more than two-thirds of the mPower sessions showed significantly greater improvements in CP-related knowledge and service utilization compared to those with lower attendance, echoing findings from other studies emphasizing the importance of consistent participation in peer-support initiatives for health behavior change [17,18,19].

Interestingly, maternal education level was not a significant predictor of CP knowledge improvement, contrasting with literature that often highlights the role of parental education in health literacy [20]. However, household size and family income were positively associated with knowledge gains, suggesting that socioeconomic factors might play a stronger role in this setting. This finding aligns with research emphasizing the complex interaction between education, socioeconomic status, and health outcomes [20,21]. Research indicates that socioeconomic disadvantage is a downstream outcome of poor educational attainment that has a significant impact on health literacy [21]. It is additionally hypothesized that socioeconomic advantage corresponds with the availability of information and having resources to obtain this information readily [22]. However, within our cohort, the accessibility of information was uniform, and having accounted specifically for educational attainment, means this cannot be the factor at play. Further research should focus on a subgroup analysis of knowledge gains to effectively determine the interplay and impact of socio-demographic factors on disease-specific knowledge.

Rehabilitation service utilization was significantly higher among mothers with higher session attendance and those with secondary education or above, consistent with studies showing that higher health literacy improves service uptake [23,24]. However, the lack of a significant association between knowledge improvement and service use indicates that additional barriers, such as healthcare accessibility or cultural factors, may influence utilization [20].

Moreover, in Bangladesh, there are a large number of untrained health practitioners, servicing rural areas of the country, particularly for populations out of reach of the government or not-for-profit health services [25,26]. Furthermore, traditional healers or untrained health practitioners are rarely equipped to provide accurate information to families about the nature of CP [26]. mPower intervention could play an important role in alleviating these knowledge and service gaps.

The effectiveness of peer-to-peer support groups for mothers of children with CP in Bangladesh is linked to the strong influence of social networks, where community leaders play a crucial role in encouraging health-seeking behaviors, even when the benefits are not immediate [27,28]. Our study highlights how improving health literacy through peer support could enhance service utilization and potentially health outcomes for children with CP in low-resource settings. Knowledgeable mothers are better equipped to understand their child’s condition, making them more effective in managing CP [29]. It means they can comprehend and implement recommended therapies, identify complications, seek early intervention, make better use of available resources, effectively use assistive devices, and make informed choices about their child’s care [30]. Knowledgeable mothers are also more likely to cope with the pressures of caring for complex medical conditions, such as CP [31]. Within this study, we used a training manual to structure our intervention; however, future programs should consider development of interventions based on detailed feedback from mothers, particularly in identifying their own gaps in knowledge and what they themselves would like to learn. Public health interventions should capitalize on the strength of a peer-to-peer approach, particularly in collectivist societies such as Bangladesh.

Our analysis of mPower attendance revealed that while maternal attributes did not significantly predict participation, the education and occupation of the partner, particularly fathers, had a substantial impact. This finding is crucial for designing effective public health interventions in low-resource settings like Bangladesh, where fathers are key decision-makers within households [32]. While most public health efforts focus on mothers, fathers’ support, particularly those with higher education and better occupations, influences how resources are mobilized for children’s health. Future iterations of the mPower program may include disseminating knowledge to fathers as well, so that they direct resources toward the health of their child. As fathers are less likely to attend rehabilitation centers due to work commitments, knowledge may be disseminated through flyers/information sheets sent home with the child. Future interventions must recognize the important role fathers play in enabling access to care for children with disabilities.

Despite our best efforts, several limitations in our study need to be considered while interpreting the findings. The lack of a control group limits the ability to attribute observed changes solely to the intervention. Furthermore, the study was conducted in a single region of Bangladesh, potentially limiting the generalizability of the findings to other low-resource settings. Attendance variability and reliance on self-reported data also introduce possible bias, and this study did not reflect on potential long-term benefits with a later follow up. Lastly, there are further opportunities to contribute to knowledge around socioeconomic characteristics through subgroup analyses. However, the use of a quasi-experimental pre-post design allowed us to directly compare mothers’ knowledge and service utilization before and after the intervention. Moreover, the use of a validated tool like the Knowledge of Cerebral Palsy Questionnaire adds rigor to the assessment. Additionally, the intervention was grounded in an existing cohort from the BCPR, ensuring relevance and context specificity.

## 5. Conclusions

The mPower intervention effectively enhanced health literacy and likely contributed to service utilization among mothers of children with CP in rural Bangladesh. Our study has demonstrated a substantial opportunity for meaningful change in the care of children with CP across LMICs. Future research should explore the long-term impacts of such interventions, the role of fathers in health decision-making, and the scalability of peer support models in diverse low-resource settings to further support and empower mothers/families of children with a disability. This study has highlighted the practical relevance of programs such as mPower across LMIC settings, and governments should invest in such programs as an effective and sustainable approach to managing children with complex healthcare needs.

## Figures and Tables

**Table 1:** Socio-demographic characteristics of the participating mothers (*N* = 289) in the mPower groups.

Respondents’ Characteristics	*N* = 289, *n* (%)
**Mothers’ education**	
Primary or lower	190 (65.7)
Secondary	73 (25.3)
Higher secondary and above	26 (9.0)
**Mothers’ occupation**	
Housewife	276 (95.5)
Job/business	13 (4.5)
**Partner’s education**	
Primary or lower	199(68.9)
Secondary	57 (19.7)
Higher secondary and above	33 (11.4)
**Partner’s occupation**	
Job/business	109 (38.1)
Agriculture/farming related work	62 (21.7)
Daily wage earners	115 (40.2)
**Household size**	
≤4	119 (41.2)
5–7	134 (46.4)
8–13	36 (12.5)
**Average monthly household income, BDT~USD ^1^**	
Mean (SD)	10,000 [10,000, 12,000]~84 [84, 100]
≤10,000 BDT (≤84 USD)	175 (61.0)
>10,000 BDT (>84 USD)	112 (39.0)
**Type of household**	
Kutcha	178 (61.6)
Semi-pucca (semi-permanent housing, some brick/concrete, tin roofing)	86 (29.8)
Pucca (permanent housing made of brick/concrete)	25 (8.7)
**Age of the child with CP**	
0–5 years	118 (41.1)
6–10 years	89 (31.0)
11–15 years	60 (20.9)
16–18 years	20 (7.0)
**Sex of the child with CP**	
Female	116 (40.1)
Male	173 (59.9)

^1^ USD~119 BDT.

**Table 2:** Factors related to improved knowledge among mPower group members.

Respondents’ Characteristics	Knowledge Improved		*p*-Value	Unadjusted Odds Ratio (OR) [CI 95%]
	No, *n* = 94, *n* (%)	Yes, *n* = 195, *n* (%)		
**Number of mPower sessions attended**				
Two-third or less	71 (36.4)	124 (63.6)	0.04	Ref
More than two-third	23 (24.5)	71 (75.5)		1.8 [1.0, 3.1]
**Mothers’ education**				
Primary or lower	66 (34.7)	124 (65.3)	0.11	Ref
Secondary	17 (23.3)	56 (76.7)		1.8 [0.9, 3.3]
Higher secondary and above	11 (42.3)	15 (57.7)		0.7 [0.3, 1.7]
**Mothers’ occupation**				
Job/business	4 (30.8)	9 (69.2)	0.58	1.1 [0.3, 3.6]
Housewife	90 (32.6)	186 (67.4)		Ref
**Partner’s education**				
Primary or lower	66 (33.2)	133 (66.8)	0.29	Ref
Secondary	21 (36.8)	36 (63.2)		0.9 [0.5, 1.6]
Higher secondary and above	7 (21.2)	26 (78.8)		1.8 [0.8, 4.5]
**Partner’s occupation**				
Job/business	30 (27.5)	79 (72.5)	0.32	Ref
Agriculture/farming related work	22 (35.5)	40 (64.5)		0.7 [0.4, 1.3]
Daily wage earners	42 (36.5)	73 (63.5)		0.7 [0.4, 1.2]
**Household size**				
≤4	49 (41.2)	70 (58.8)	0.03	Ref
5–7	41 (27.3)	109 (72.7)		1.9 [1.1, 3.1]
>7	4 (20.0)	16 (80.0)		2.8 [0.9, 8.9]
**Monthly household income**				
≤10,000 BDT (≤84 USD)	66 (37.7)	109 (62.3)	0.03	Ref
>10,000 BDT (>84 USD)	28 (25.0)	84 (75.0)		1.8 [1.1, 3.1]

**Table 3:** Factors related to accessing services at rehabilitation centers for their children.

Respondents’ Characteristics	Frequency of Attending Centre		*p*-Value	Unadjusted Odds Ratio (OR) [CI 95%]
	≤12 Times, *n* = 193, *n* (%)	>12 Times, *n* = 96, *n* (%)		
**Mothers’ education**				
Primary or lower	135 (71.1)	55 (28.9)	0.001	Ref
Secondary	49 (67.1)	24 (32.9)		1.2 [0.7, 2.1]
Higher secondary and above	9 (34.6)	17 (65.4)		4.6 [1.9, 11.0]
**Mothers’ occupation**				
Housewife	182 (65.9)	94 (34.1)	0.14	Ref
Job/business	11 (84.6)	2 (15.4)		0.4 [0.1, 1.6]
**Partner’s education**				
Primary or lower	143 (71.9)	56 (28.1)	0.003	Ref
Secondary	36 (63.2)	21 (36.8)		1.5 [0.8, 2.8]
Higher secondary and above	14 (42.4)	19 (57.6)		3.5 [1.6, 7.4]
**Partner’s occupation**				
Job/business	63 (57.8)	46 (42.2)	0.05	1.7 [1.0 3.0]
Agriculture/farming related work	46 (74.2)	16 (25.8)		0.8 [0.4, 1.7]
Daily wage earners	81 (70.4)	34 (29.6)		Ref
**Household size**				
≤4	80 (67.2)	39 (32.8)	0.80	Ref
5–7	101 (67.3)	49 (32.7)		1.0 [0.6, 1.7]
>7	12 (60.0)	8 (40.0)		1.4 [0.5, 3.6]
**Monthly household income**				
≤10,000 BDT (≤84 USD)	120 (68.6)	55 (31.4)	0.45	Ref
>10,000 BDT (>84 USD)	72 (64.3)	40 (35.7)		1.2 [0.7, 2.0]
**Number of mPower sessions attended**				
Two-thirds or less	151 (77.4)	44 (22.6)	<0.001	Ref
More than two-thirds	42 (44.7)	52 (55.3)		4.2 [2.5, 7.2]
**Improved knowledge about CP**				
No	66 (70.2)	28 (29.8)	0.39	Ref
Yes	127 (65.1)	68 (34.9)		1.3 [0.7, 2.1]

**Table 4:** Predictors of mother’s attendance to mPower sessions, their knowledge improvement about CP, and rehabilitation service utilization by their children.

Characteristics	Attended > Two-Third mPower Sessions	Knowledge About CP Improved	Sought Rehabilitation Services > 12 Times
	aOR [95% CI]	aOR [95% CI]	aOR [95% CI]
**Mothers’ education**			
Primary or lower	3.4 [1.0, 11.3]	2.7 [0.8, 9.0]	0.2 [0.0, 0.6]
Secondary	1.4 [0.5, 4.4]	4.5 [1.3, 15.5]	0.3 [0.1, 0.8]
Higher secondary and above	Ref	Ref	Ref
**Mothers’ occupation**			
Housewife	Ref	Ref	Ref
Job/business	0.7 [0.2, 2.8]	1.4 [0.4, 5.0]	0.3 [0.0, 1.3]
**Partner’s education**			
Primary or lower	Ref	Ref	Ref
Secondary	3.2 [1.5, 6.8]	0.5 [0.2, 1.1]	1.0 [0.4, 2.2]
Higher secondary and above	3.8 [1.3, 11.7]	1.7 [0.5, 6.3]	1.3 [0.4, 4.1]
**Partner’s occupation**			
Job/business	1.4 [0.7, 2.7]	1.3 [0.7, 2.7]	1.1 [0.5, 2.2]
Agriculture/farming related work	1.4 [0.7, 2.8]	0.9 [0.5, 1.8]	0.7 [0.3, 1.5]
Daily wage earners	Ref	Ref	Ref
**Household size**			
≤4	Ref	Ref	Ref
5–7	1.4 [0.8, 2.5]	1.7 [1.0, 3.0]	0.6 [0.4, 1.2]
>7	0.9 [0.3, 2.7]	3.1 [0.9, 10.6]	1.1 [0.4, 3.3]
**Monthly household income**			
≤10,000 BDT (≤84 USD)	Ref	Ref	Ref
>10,000 BDT (>84 USD)	0.9 [0.5, 1.6]	1.5 [0.9, 2.7]	1.0 [0.5, 1.8]
**Number of mPower sessions attended**			
Two-third or less	-	Ref	Ref
More than two-third	-	1.9 [1.0, 3.4]	4.7 [2.6, 8.5]
**Knowledge about CP improved**			
No	-	-	Ref
Yes	-	-	1.1 [0.6, 2.1]

## Data Availability

The data supporting the conclusions of this article will be made available by the authors on request.

## Supplementary Materials

The following supporting information can be downloaded at: https://www.mdpi.com/article/10.3390/children11121438/s1, Supplementary Table S1: Factors related to mother’s attendance to the mPower sessions.

## References

[1] Rosenbaum P., Paneth N., Leviton A., Goldstein M., Bax M., Damiano D., Dan B., Jacobsson B. (2007). A report: The definition and classification of cerebral palsy April 2006. Dev. Med. Child Neurol. Suppl..

[2] Sadowska M., Sarecka-Hujar B., Kopyta I. (2020). Cerebral Palsy: Current Opinions on Definition, Epidemiology, Risk Factors, Classification and Treatment Options. Neuropsychiatr. Dis. Treat..

[3] Khandaker G., Muhit M., Karim T., Smithers-Sheedy H., Novak I., Jones C., Badawi N. (2019). Epidemiology of cerebral palsy in Bangladesh: A population-based surveillance study. Dev. Med. Child Neurol..

[4] World Health Organization (1998). Health Promotion Glossary. https://iris.who.int/bitstream/handle/10665/64546/WHO_HPR_HEP_98.1.pdf?sequence=1.

[5] Berkman N.D., Sheridan S.L., Donahue K.E., Halpern D.J., Crotty K. (2011). Low health literacy and health outcomes: An updated systematic review. Ann. Intern. Med..

[6] Perrins G., King C., Jahan I., Hashan R., Azhdari K., Power R., Badawi N., Khandaker G. Health literacy of primary caregivers of children with cerebral palsy in low- and middle-income countries: A systematic review. BMJ Open.

[7] DeWalt D.A., Hink A. (2009). Health literacy and child health outcomes: A systematic review of the literature. Pediatrics.

[8] Al Imam M.H., Jahan I., Muhit M., Hardianto D., Laryea F., Chhetri A.B., Smithers-Sheedy H., McIntyre S., Badawi N., Khandaker G. (2021). Predictors of Rehabilitation Service Utilisation among Children with Cerebral Palsy (CP) in Low- and Middle-Income Countries (LMIC): Findings from the Global LMIC CP Register. Brain Sci..

[9] Damon W. (1984). Peer education: The untapped potential. J. Appl. Dev. Psychol..

[10] Nalugya R., Nambejja H., Nimusiima C., Kawesa E.S., van Hove G., Seeley J., Bannink M.F. (2023). Obuntu bulamu: Parental peer-to-peer support for inclusion of children with disabilities in Central Uganda. Afr. J. Disabil..

[11] Bray L., Carter B., Sanders C., Blake L., Keegan K. (2017). Parent-to-parent peer support for parents of children with a disability: A mixed method study. Patient Educ. Couns..

[12] Lancaster K., Bhopti A., Kern M.L., Taylor R., Janson A., Harding K. (2023). Effectiveness of peer support programmes for improving well-being and quality of life in parents/carers of children with disability or chronic illness: A systematic review. Child Care Health Dev..

[13] Khandaker G., Smithers-Sheedy H., Islam J., Alam M., Jung J., Novak I., Booy R., Jones C., Badawi N., Muhit M. (2015). Bangladesh Cerebral Palsy Register (BCPR): A pilot study to develop a national cerebral palsy (CP) register with surveillance of children for CP. BMC Neurol..

[14] CSF Global Getting to Know Cerebral Palsy. https://csf-global.org/wp-content/uploads/2015/11/Getting-to-know-cerebral-palsy-v1-lowres.pdf.

[15] Perrins G., Jahan I., Islam S., Al Imam M.H., King C., Muhit M., Badawi N., Khandaker G. mPower (mother’s power): A peer support initiative for mothers of children with cerebral palsy in rural Bangladesh. JMIR Form. Res..

[16] Dambi J.M., Mandizvidza C., Chiwaridzo M., Nhunzvi C., Tadyanemhandu C. (2016). Does an educational workshop have an impact on caregivers’ levels of knowledge about cerebral palsy? A comparative, descriptive cross-sectional survey of Zimbabwean caregivers. Malawi Med. J..

[17] Chakraborti M., Gitimoghaddam M., McKellin W.H., Miller A.R., Collet J.-P. (2021). Understanding the Implications of Peer Support for Families of Children With Neurodevelopmental and Intellectual Disabilities: A Scoping Review. Front. Public Health.

[18] Shilling V., Bailey S., Logan S., Morris C. (2015). Peer support for parents of disabled children part 1: Perceived outcomes of a one-to-one service, a qualitative study. Child Care Health Dev..

[19] Kingsnorth S., Gall C., Beayni S., Rigby P. (2011). Parents as transition experts? Qualitative findings from a pilot parent-led peer support group. Child Care Health Dev..

[20] Zaidman E.A., Scott K.M., Hahn D., Bennett P., Caldwell P.H. (2023). Impact of parental health literacy on the health outcomes of children with chronic disease globally: A systematic review. J. Paediatr. Child Health.

[21] Nutbeam D., Lloyd J.E. (2021). Understanding and Responding to Health Literacy as a Social Determinant of Health. Annu. Rev. Public Health.

[22] Stormacq C., Van den Broucke S., Wosinski J. (2019). Does health literacy mediate the relationship between socioeconomic status and health disparities? Integrative review. Health Promot. Int..

[23] Levy H., Janke A. (2016). Health Literacy and Access to Care. J. Health Commun..

[24] de Buhr E., Tannen A. (2020). Parental health literacy and health knowledge, behaviours and outcomes in children: A cross-sectional survey. BMC Public Health.

[25] World Health Organization (2015). Bangladesh Health System Review. https://iris.who.int/handle/10665/208214.

[26] Das S., Mia M.N., Hanifi S.M.A., Hoque S., Bhuiya A. (2017). Health literacy in a community with low levels of education: Findings from Chakaria, a rural area of Bangladesh. BMC Public Health.

[27] Miller G., Mobarak M. (2023). Marketing of Stoves through Social Networks to Combat Indoor Air Pollution. https://www.theigc.org/publications/marketing-stoves-through-social-networks-combat-indoor-air-pollution-policy-brief.

[28] Frank L.B., Jodrell D., Smethurst L. (2017). Social and structural factors to promote maternal health in Bangladesh. J. Commun. Healthc..

[29] He C., Evans N., Graham H. (2024). Group-based caregiver support interventions for children living with disabilities in low-and- middle-income countries: Narrative review and analysis of content, outcomes, and implementation factors. J. Glob. Health.

[30] World Health Organization Global Report on Children with Developmental Disabilities. https://www.who.int/publications/i/item/9789240080539.

[31] Power R., Muhit M., Heanoy E., Karim T., Badawi N., Khandaker G. (2021). Depression, anxiety and stress among caregivers of adolescents with cerebral palsy in rural Bangladesh. Disabil. Rehabil..

[32] Jeong J., Sullivan E.F., McCann J.K. (2023). Effectiveness of father-inclusive interventions on maternal, paternal, couples, and early child outcomes in low- and middle-income countries: A systematic review. Soc. Sci. Med..

