# Supplementary material for: The mPower (Mother’s Power) Initiative: Improving Health Behavior Through Peer Support and Health Literacy for Mothers of Children with Cerebral Palsy in Rural Bangladesh
Source: Children (Basel). 2024 Nov 26;11(12):1438. doi: 10.3390/children11121438 (PMC11674198; doi:10.3390/children11121438)
Supplement: Supplementary file 1 [file children-11-01438-s001.zip › children-3286124-Supplementary.pdf]

Supplementary Table S1. Factors related to mother's attendance to the mPower sessions.

Respondents' characteristics	Number of mPower sessions attended		p-value[1]	Unadjusted odds ratio (OR)
	Two-third or less, n=195, n (%)	More than two-third, n=94, n (%)		
**Mothers' education**				
Primary or lower	125 (65.8)	65 (34.2)	0.687	1.2 [0.5, 2.8]
Secondary	52 (71.2)	21 (28.8)		0.9 [0.3, 2.4]
Higher secondary and above	18 (69.2)	8 (30.8)		Ref
**Mothers' occupation**				
Housewife	185 (67.0)	91 (33.0)	0.457	Ref
Job/ business	10 (76.9)	3 (23.1)		0.61 [0.16-2.3]
**Partner's education**				
Primary or lower	145 (72.9)	54 (27.1)	0.014	Ref
Secondary	19 (57.6)	14 (42.4)		**2.3 [1.2, 4.1]**
Higher secondary and above	31 (54.4)	26 (45.6)		2.0 [0.9, 4.2]
**Partner's occupation**				
Job/business	66 (60.6)	43 (39.4)	0.102	**1.8 [1.0, 3.3]**
Agriculture/farming related work	41 (66.1)	21 (33.9)		1.5 [0.7, 2.8]
Daily wage earners	85 (73.9)	30 (26.1)		Ref
**Household size**				
≤4	87 (73.1)	32 (26.9)	0.161	Ref
5-7	83 (61.9)	51 (38.1)		1.6 [1. 0-2.7]
>7	25 (69.4)	11 (30.6)		1.2 [0.4-3.3]
**Monthly household income**				
≤ 10000 BDT (≤ 84 USD)	119 (68.9)	56 (32.0)	0.855	Ref
>10000 BDT (> 84 USD)	75 (67.0)	37 (33.0)		1.0 [0.6, 1.7]

[1]Chi-quared test
